# Exceptional Seizure-Like Presentation of Torsades De Pointes

**DOI:** 10.7759/cureus.77403

**Published:** 2025-01-13

**Authors:** Kanchan Kanchan, Ahmed Mohamed, Pawan Kumar

**Affiliations:** 1 Cardiology, University Hospitals Birmingham, Birmingham, GBR; 2 General Internal Medicine, University Hospitals Birmingham, Birmingham, GBR

**Keywords:** cardiac arrhythmia, coronary stenosis, premature ventricular contractions, r-on-t phenomenon, seizure-like episode, torsade’s de pointes, ventricular tachycardia

## Abstract

We present a rare case of a 40-year-old male who experienced a seizure-like episode, later diagnosed as torsades de pointes (TDP), following an R-on-T phenomenon feature on ECG. This case underscores the importance of considering cardiac arrhythmias in the differential diagnosis of atypical seizure presentations. The patient underwent successful treatment with antiarrhythmics and coronary stenting for underlying coronary artery stenosis, emphasizing a comprehensive approach to managing such complex cases.

## Introduction

Torsades de pointes (TDP) is a form of polymorphic ventricular tachycardia (VT) characterized by a twisting QRS complex on the electrocardiogram (ECG) [1]. Typically associated with prolonged QT intervals, TDP can also occur with normal QT intervals, particularly in the presence of short coupled premature ventricular contractions (PVCs) [2,3] and following an R-on-T phenomenon, which is the superimposition of an ectopic beat on the T wave of a preceding beat [4-6]. TDP commonly presents as syncope or sudden cardiac arrest but rarely as seizure-like episodes due to transient cerebral hypoperfusion [7-9]. This case illustrates the challenges and critical considerations in diagnosing and managing TDP presenting as a seizure-like episode.

## Case presentation

A 40-year-old Caucasian male experienced a two- to three-minute episode of generalized shaking witnessed by his girlfriend without predisposing factors or typical seizure signs such as tongue biting or incontinence. The episode self-resolved, and he felt as though he had awoken from a long sleep with no postictal confusion but significant sleepiness afterward. He has no significant medical history, takes no regular medications, and has no family history of cardiac disease. Socially, he is a non-smoker, consumes alcohol occasionally, and does not use recreational drugs. On examination, his vital signs were normal, alert, and oriented, and his cardiovascular and neurological assessments were unremarkable, showing no signs of postictal state or acute distress. 

Diagnostic assessment

**Figure 1:**
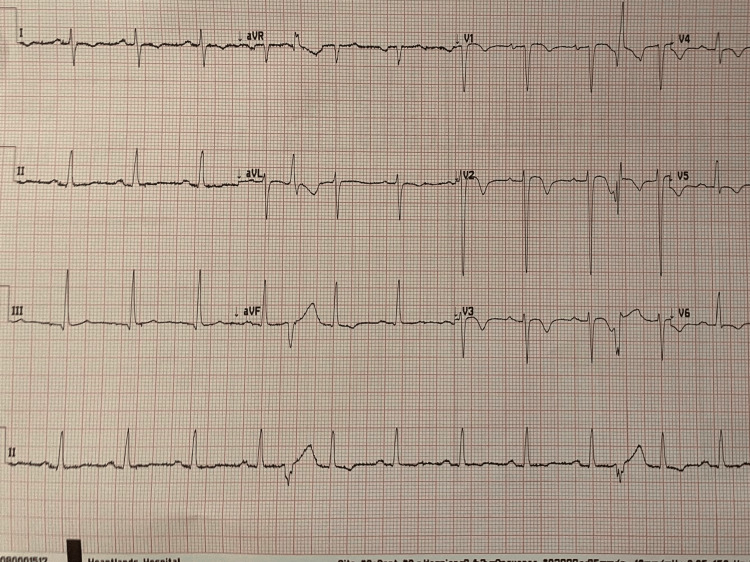
The ECG demonstrates a premature ventricular complex (PVC) occurring on the descending limb of the T wave, consistent with the R-on-T phenomenon.

**Figure 2:**
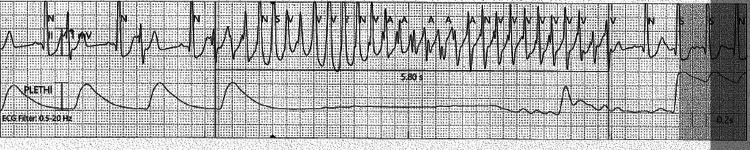
The ECG demonstrates a premature ventricular contraction (PVC) occurring on the descending limb of the T wave (R-on-T phenomenon). This triggers a polymorphic ventricular tachycardia consistent with torsades de pointes, characterized by twisting QRS complexes with varying amplitude and morphology.

**Figure 3:**
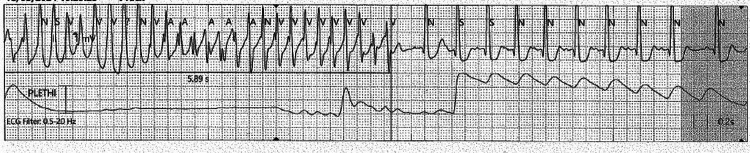
ECG demonstrates non-sustained polymorphic ventricular tachycardia (VT).

**Figure 4:**
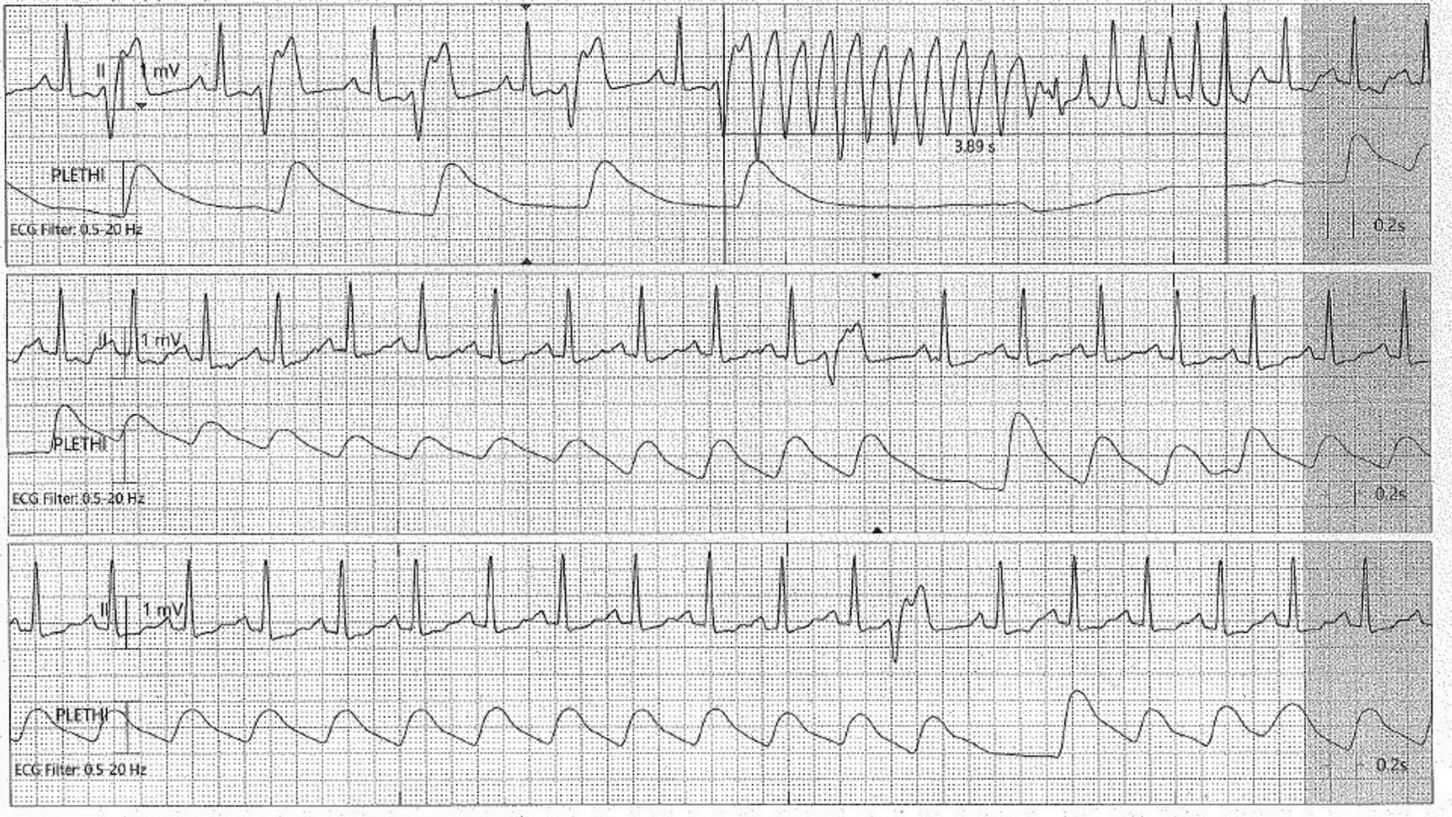
ECG demonstrates premature ventricular contraction (PVC) with R-on-T phenomenon leading to non-sustained ventricular tachycardia (VT) in the form of torsades de pointes.

Upon initial diagnostic assessment, he exhibited short-coupled PVCs on an ECG with an R-on-T phenomenon (Figures 1-2), with a normal QTc interval of 430 ms [2,5,6]. Laboratory tests, including a complete blood count showing normal findings, serum potassium levels at 4.2 mmol/L and magnesium at 1.9 mg/dL, negative troponin I, and normal thyroid function, provided no indications of acute metabolic or myocardial abnormalities. Cardiac monitoring revealed non-sustained episodes of TDP (Figure 3) alongside persistent ventricular ectopy (VEs) and episodes of VT (Figure 4) requiring direct current cardioversion (DCCV) [4,10]. Coronary angiogram findings indicated moderate stenosis in the mid-segment of the right coronary artery (RCA), with catheter spasm hindering further intervention and tight stenosis in the mid-segment of the left anterior descending (LAD) artery. Subsequent cardiac magnetic resonance imaging (MRI) revealed severe hypokinesia in the mid and apical septum extending to the apex, as well as hypokinesia in the basal anterior wall indicative of infarcts in the LAD territory [11,12]. This resulted in moderately impaired left ventricular (LV) systolic function, with an ejection fraction measured at 38%. The cardiac valves and right heart function were normal.

## Discussion

Pathophysiology

TDP is typically linked to prolonged QT intervals. However, in this case, the presence of an R-on-T phenomenon and short-coupled PVCs, despite a normal QTc, posed a significant risk for TDP. Myocardial ischemia likely exacerbated the electrical instability, precipitating the TDP episodes [5]. This case emphasizes the need to consider TDP in patients with normal QT intervals who exhibit an R-on-T phenomenon and short-coupled PVCs and have a potential ischemic trigger [9]. 

Clinical implications

This case demonstrates the diagnostic complexity when TDP presents as a seizure-like episode, particularly in the absence of typical postictal symptoms and a normal QTc interval. It highlights the importance of considering cardiac etiologies such as TDP in the differential diagnosis of seizure-like episodes, especially in patients with no prior neurological history [1,2]. 

Management strategies

The successful management of TDP in this case involved prompt identification and treatment with antiarrhythmic and revascularization of coronary artery stenosis. The patient’s outcome illustrates the importance of a multidisciplinary approach in the diagnosis and treatment of complex cases involving atypical presentations of cardiac arrhythmias [5-7].

Management and complication

In the acute management phase, the patient initially received intravenous amiodarone with a dose of 300 mg, followed by a maintenance dose of 900 mg, along with intravenous metoprolol for rate control. Interventions included multiple sessions of DCCV [5] due to recurrent episodes of VT and TDP. Additionally, a temporary pacing wire (TPW) was inserted, maintaining pacing at 100 bpm to mitigate further TDP occurrences [5,10]. For long-term management, the patient underwent percutaneous coronary intervention (PCI) with placement of a drug-eluting stent (DES) sized 3.0 × 20 mm in the LAD artery to address tight stenosis [4,12]. The patient experienced a ventricular fibrillation (VF) episode, necessitating immediate intervention. Cardiopulmonary resuscitation (CPR) was initiated according to advanced life support (ALS) protocols, successfully reviving the patient [10]. For secondary prevention of TDP and VF, a subcutaneous implantable cardioverter-defibrillator (ICD) [6,13,14] was recommended to mitigate the risk of future life-threatening arrhythmias. 

## Conclusions

This case underscores the necessity of considering cardiac arrhythmias, particularly TDP, in the differential diagnosis of seizure-like episodes. Comprehensive cardiac evaluation and timely intervention are paramount to prevent potentially fatal outcomes. The case also highlights the role of an R-on-T phenomenon, short-coupled PVCs, and myocardial ischemia as risk factors for TDP, even in the presence of a normal QT interval.
